# EUREST-RISE: An innovative networking and training project on European Tobacco Control

**DOI:** 10.18332/tpc/163137

**Published:** 2023-04-24

**Authors:** Cornel Radu-Loghin, Karina Mocanu, Hani Al Gouhmani, Constantine Vardavas, Ioanna Lagou, Zinovia Plyta, Aikaterini Papathanasaki, Stella Vogiatzidaki, Alexander Vardavas, Manolis Tzatzarakis, Aristidis Tsatsakis, Filippos Filippidis, Christina Kyriakos, Esteve Fernandez, Olena Tigova, Cristina Martinez, Anna Mar Lopez Luque, Marius Eremia, Lucia Maria Lotrean, Antigona Trofor, Thomas Wenzl, Bill Simpson, Pippa Powell, Polina Starchenko, Angeliki Bakou, Eleni Asimaki, Victoria Vivilaki

**Affiliations:** 1 European Network for Smoking and Tobacco Prevention, Brussels, Belgium; 2 School of Medicine, University of Crete, Heraklion, Greece; 3 Imperial College London, London, United Kingdom; 4 Catalan Institute of Oncology, Spain; 5 Bellvitge Biomedical Research Institute, Spain; 6 School of Medicine and Health Sciences, Universitat de Barcelona, Barcelona, Spain; 7 CIBER of Respiratory Diseases, Spain; 8 Aer Pur Romania, Romania; 9 Joint Research Centre/European Commission; 10 CARA Technology Ltd, United Kingdom; 11 European Lung Foundation, Brussels, Belgium; 12 University of West Attica, Athens, Greece

**Keywords:** EUREST-RISE, tobacco control, training project

The design and additives of tobacco products are significant factors that facilitate industry innovations that may undermine public health programs and contribute to tobacco use and addiction^1,2^. Although nicotine and its pharmacological effects are central for sustaining tobacco product use, they may be insufficient to support product acceptance, as sensory stimuli attributable to flavors are particularly critical, reducing subjective measures of craving and withdrawal^3,4^. Therefore, in recent years, the tobacco industry has developed and introduced various cigarette technologies and design features, including new tobacco blends, additives, and, subsequently, new flavors. While the importance of flavors has been researched by the tobacco industry in the context of product development, the assessment of flavors for regulatory purposes with the use of sensory and chemical analyses is still under development – hence an emerging area for incoming tobacco control researchers^5^.

Furthermore, novel tobacco products that further perpetuate the complexity of understanding and effectively regulating products have emerged, and hence tobacco control research now covers a breadth of products. Like the breadth of COVID-19 variants encountered during the pandemic – each tobacco product has a different epidemiology and different ‘high-risk groups’, but all can be appropriately addressed with the application of specific surveillance and mitigation measures.

In response to this new reality in tobacco control, the EUREST-RISE Project was launched with funding under the Marie Skłodowska-Curie Action for Staff Exchanges^6^. The project proposes a series of integrated studies that were proposed to facilitate the training of researchers to examine tobacco product systems, their mechanisms and purpose, interactions between key design characteristics and their assessment, and monitoring in the population. The research training facilitated within EUREST-RISE will be coupled with training on communicating to key stakeholders, among others, policymakers, the media and the general public.

With a consortium of academic and non-academic partner organizations, the EUREST-RISE project has three operational pillars, each of them directly linked to one of the vertical Work Packages (WPs).

1. WP2 aims to facilitate the exchange of expertise on complex dataset analysis and interpretation at both the product and population level through the analysis of European Union Common Entry Gate (EU-CEG) product-level data and available European population-based secondary datasets.
2. WP3 supports the exchange of expertise on the sensory and chemical analysis of tobacco, e-cigarettes and novel products, including training and research on chemical and toxicological assessment.
3. WP4 supports training in advocacy, policy integration and communication on tobacco control with training in peer-to-peer, media, and stakeholder communication.

The complex and multidisciplinary nature of tobacco product regulation involving public health, epidemiology, toxicology, chemistry, sensory science, and public policy, are constructs of training which have yet to be formulated into any formal academic training process in the EU. It is this gap that the EUREST-RISE project will cover through the creation of a cohort of researchers who will be trained as scholars on state-of-the-art aspects of European tobacco product regulatory science.

## Data Availability

Data sharing is not applicable to this article as no new data were created.
